# Supplementary material for: EnzML: multi-label prediction of enzyme classes using InterPro signatures
Source: BMC Bioinformatics. 2012 Apr 25;13:61. doi: 10.1186/1471-2105-13-61 (PMC3483700; doi:10.1186/1471-2105-13-61)
Supplement: Addtional file 5 — The Java code to format the data files, evaluate and predict. The file enzml_java_code.tar.gz contains the Java code used to format database data to ARFF and XML formats, to execute cross and train-test (jackknife) evaluations and to record evaluation results to database. More information is included in the readme.txt file and the Javadoc files. The code can be used with a MySQL database. To use a different database software, other JDBC drivers might be required. [file 1471-2105-13-61-S5.gz › java_code/utils/doc/uk/ac/ed/inf/utils/PathUtils.html]

PathUtils


---


|  |  |  |
| --- | --- | --- |
| **Overview** \| **Package** \| **Class** \| **Use** \| **Tree** \| **Deprecated** \| **Index** \| **Help** | |  |
| **PREV CLASS**   **NEXT CLASS** | **FRAMES**    **NO FRAMES**     **All Classes** |  |
| SUMMARY: NESTED \| FIELD \| CONSTR \| METHOD | DETAIL: FIELD \| CONSTR \| METHOD |  |


---


## uk.ac.ed.inf.utils Class PathUtils

```
java.lang.Object
  uk.ac.ed.inf.utils.PathUtils
```

---

``` public class PathUtils extends java.lang.Object ```

Paths to utilities folders and files

**Version:**
:   24 Feb 2009

**Author:**
:   Luna De Ferrari luna.deferrari-at-ed.ac.uk

---

| **Field Summary** | |
| --- | --- |
| `static java.lang.String` | `DATA_DB_CONN_PATH` |
| `static java.lang.String` | `DB_UTILS_HOME` |
| `static java.lang.String` | `TEST_DB_CONN_PATH` |
| `static java.lang.String` | `TEST_HOME` |
| `static java.lang.String` | `UTILS_DATA` |
| `static java.lang.String` | `UTILS_HOME` |


**Constructor Summary**

`PathUtils()`


**Method Summary**

**Methods inherited from class java.lang.Object**

`equals, getClass, hashCode, notify, notifyAll, toString, wait, wait, wait`

**Field Detail**

### UTILS\_HOME

```
public static java.lang.String UTILS_HOME
```

---


### TEST\_HOME

```
public static java.lang.String TEST_HOME
```

---


### UTILS\_DATA

```
public static java.lang.String UTILS_DATA
```

---


### DB\_UTILS\_HOME

```
public static java.lang.String DB_UTILS_HOME
```

---


### TEST\_DB\_CONN\_PATH

```
public static java.lang.String TEST_DB_CONN_PATH
```

---


### DATA\_DB\_CONN\_PATH

```
public static java.lang.String DATA_DB_CONN_PATH
```


**Constructor Detail**

### PathUtils

```
public PathUtils()
```


---


|  |  |  |
| --- | --- | --- |
| **Overview** \| **Package** \| **Class** \| **Use** \| **Tree** \| **Deprecated** \| **Index** \| **Help** | |  |
| **PREV CLASS**   **NEXT CLASS** | **FRAMES**    **NO FRAMES**     **All Classes** |  |
| SUMMARY: NESTED \| FIELD \| CONSTR \| METHOD | DETAIL: FIELD \| CONSTR \| METHOD |  |


---
